# Investigating a Commercial Functional Adhesive with 12-MDPB and Reactive Filler to Strengthen the Adhesive Interface in Eroded Dentin

**DOI:** 10.3390/polym13203562

**Published:** 2021-10-15

**Authors:** Madalena Belmar da Costa, António HS Delgado, Tomás Amorim Afonso, Luís Proença, Ana Sofia Ramos, Ana Mano Azul

**Affiliations:** 1 Unit of Conservative Dentistry, Instituto Universitário Egas Moniz (IUEM), Monte de Caparica, 2829-511 Almada, Portugal; madalenabelmarc@gmail.com (M.B.d.C.); tamorim.tervis@gmail.com (T.A.A.); aazul@egasmoniz.edu.pt (A.M.A.); 2 Centro de Investigação Interdisciplinar Egas Moniz (CiiEM), Monte de Caparica, 2829-511 Almada, Portugal; lproenca@egasmoniz.edu.pt; 3 Division of Biomaterials & Tissue Engineering, UCL Eastman Dental Institute, Royal Free Hospital, Hampstead, London NW3 2PF, UK; 4 Department of Mechanical Engineering, University of Coimbra, CEMMPRE, 3030-788 Coimbra, Portugal; sofia.ramos@dem.uc.pt

**Keywords:** biomaterials, dental adhesive, dental erosion, eroded dentin, resin-dentin interface

## Abstract

To compare the adhesive interface of eroded dentin formed by a functional dental adhesive and a gold standard strategy, by testing microtensile bond strength (μTBS), hardness/elastic modulus. Permanent sound human molars were randomly allocated to four experimental groups, all subject to artificial erosion (0.05 M citric acid; 3× daily, 5 days). Groups included control Clearfil SE Bond 2 (CFSE), and experimental group Clearfil SE Protect (CFP), at two different time points-immediate (24 h) and long term (3 months–3 M). Samples were sectioned into microspecimens for μTBS (*n* = 8) and into 2-mm thick slabs for nanoindentation assays (*n* = 3). Groups CFSE_3M and CFP_3M were stored in artificial saliva. Statistical analysis included two-way ANOVA for μTBS data, while hardness/modulus results were analyzed using Kruskal–Wallis H Test (significance level of 5%; SPSS v.27.0). Although no significant differences were found between mean μTBS values, for different adhesives and time points (*p* > 0.05), a positive trend, with μTBS rising in the CFP_3M group, was observed. Regarding hardness, no significant differences were seen in the hybrid layer, considering the two variables (*p* > 0.05), while the reduced elastic modulus rose in CFP_3M when compared to 24 h. Thus, CFP shows similar mechanical and adhesive performance to CFSE in eroded dentin, although it may comprise promising long-term results. This is advantageous in eroded substrates due to their increased enzymatic activity and need for remineralization.

## 1. Introduction

Dental erosion is a prevalent condition, globally, and is suffering a significant increase in the last few decades [1]. Not only is this condition more common as age increases, but it also affects a younger population, which is rising in number [2]. Erosion causes several challenges from its diagnosis through to its treatment and resolution [2,3]. Adhesive dentistry techniques are, therefore, the preferred current method to conserve substrates, while procuring biomimetic restorative options in these cases [4,5].

The single most important concern in dental adhesion, currently, is treatment longevity [5,6]. Despite the technology advancements over the past years, with novel materials, surface pre-treatments, simplified systems and adhesive strategies being developed, the hybrid layer formed in dentin is still persistently described as the weakest link in the restorative complex [7,8,9]. Thus, in the eroded substrate, creating, managing and stabilizing this hybrid layer is made more arduous [4,9,10]. Erosion is responsible for causing severe, or occasionally, irreversible damage to the mineral phases in enamel and dentin [2,4]. Due to the varying microstructural alterations that occur in eroded enamel and dentin, the choice of adhesive system gains a considerable weight in the success of the treatment [4,5].

Current findings suggest that eroded enamel is a beneficial substrate to bond to [11,12,13]. However, with eroded dentin there is a complex challenge to resolve [4]. Occluded tubules and the presence of a hypermineralized layer are factors which affect bonding properties and overall treatment success [10,12]. Additionally, this altered structure does not allow sufficient monomer penetration and infiltration across the surface, weakening bond strength [9]. With a less thick and stable hybrid layer, its integrity becomes compromised [14]. The adhesive interface in such cases is also reported to be more susceptible to microleakage, hydrolytic and enzymatic degradation, contributing to the failure of a long-term treatment [9,14,15]. Despite the existence of studies which have tried to overcome the hardships when bonding to eroded dentin, consensus on effective protocols is yet to be reached [4,9,16].

Past research has revealed that formulations containing 10-methacryloyloxydecyl dihydrogen phosphate (10-MDP) seem to form stable interfaces [17]. This monomer is not only able to create hybrid layers which are more stable, over time, but also leads to better bond strength results [4,5,7,9,17]. In fact, in eroded dentin, the inorganic matrix is partially demineralized, leading to more calcium present, able to interact with 10-MDP, providing more chemical bonding mechanism effect [18,19]. Moreover, self-etch adhesives that benefit from fluoride release, are also able to promote conditions that lead to the formation of an acid-base resistant zone–ABRZ, as it is described [20]. This layer, formed after erosion processes, stimulated by the constant release of fluoride at the interface, has proven to be strengthen the hybrid layer, by conferring interesting nanomechanical properties to it [20,21]. In fact, ABRZ provides a reinforced hybrid layer, capable of better resisting future acidic challenges [20,21]. However, to the best of our knowledge, such strategies have not yet been researched in already eroded substrates, nor long-term results combined with bond strength determination [22].

Clearfil SE Protect, a commercial functional adhesive, not only contains functional monomer 10-MDP, but also an encapsulated sodium fluoride reactive particle, able to release fluoride content over the time, without compromising the integrity of the adhesive interface [23,24]. It also contains the functional monomer 12-methacryloyloxydodecylpyridinium bromide (MDPB). This polymerizable quaternary ammonium methacrylate is incorporated into self-etching primers due to its strong antimicrobial properties, present before and after curing, that do not seem to compromise bond strength results [24]. Moreover, MDPB may also inhibit collagenolytic enzymes, thus protecting the hybrid layer [23,24]. Therefore, this adhesive seems to gather the conditions to form a more resistant and reinforced hybrid layer, able to protect eroded dentin from further damage [21].

Literature has proven that eroded dentin presents lower bond strength results when compared to sound dentin [4,9,10]. In summary, to combat the challenge of dental erosion which includes higher susceptibility to degradation and enzymatic activity, together with an affected, demineralized substrate, a functional material which can intervene in both is required [4]. Recent studies considering 12-MDPB and Clearfil Protect SE have focused on its antibacterial activity and lack long-term results [25]. Moreover, studies that researched this commercial adhesive in eroded dentin have reported findings related to its protective effect before erosion processes, rather than the effect on the long-term properties after erosion [26,27].

Thus, to study the effects of a functional monomer and reactive particle on eroded dentine, two adhesives were compared in this paper. Clearfil SE Bond serves as control, whereas Clearfil SE Protect is the experimental group, both tested on immediately (24 h) and long term. The aim of this study was thus to investigate the influence of the use of a functional adhesive in eroded dentin by testing the adhesive interface formed. The null hypotheses were: (1) there are no differences in the microtensile bond strength of the functional adhesive, when compared to the control, immediately and after 3 months of aging, and (2) there are no differences in the hardness and reduced elastic modulus, immediately and also after 3 months of aging.

## 2. Materials and Methods

### 2.1. Selection and Preparation of Teeth and Materials

For this in vitro study, forty-four recently extracted, permanent and non-carious human molars were selected. These were free of caries, restorations or structural defects, and obtained with consent, and under approval by the Ethics Committee of Instituto Universitário Egas Moniz (Protocol no. 938). After scaling and cleaning, the teeth were stored in 1% Chloramine T (*v*/*v*) at 4 °C, for five days and then stored in distilled water until they were used. All the molars were used within 6 months after extraction [28]. The teeth were firstly sectioned parallel to the occlusal surface, using a low-speed diamond saw (Accutom-50, Struers A/S, Ballerup, Denmark), operating at 0.350 mm/s, under water cooling, thus exposing the mid-coronal dentin.

The materials used throughout this study, and their composition, according to the manufacturers are shown in Table 1.

### 2.2. Erosive Challenge

The specimens were cyclically and artificially demineralized and remineralized. Three pH cycles, per day, at room temperature (22 °C were held on all forty-four specimens, for five consecutive days, according to previously published protocols [9,11,16].

The sectioned teeth were eroded by demineralization protocol using citric acid (0.05 M, pH 3.7 ± 0.1) for five minutes (10 mL per teeth), under constant agitation (170 rpm) using an agitation plate, and then rinsed for 5 s with distilled water. To simulate the remineralization process, the teeth were stored in artificial saliva, prepared according to the protocol previously published [16] (0.4 g NaCl, 0.4 g KCl, 0.906 g CaCl_2_·H_2_O, 0.39 g NaH_2_PO_4_·H_2_O, 0.142 g NaHPO_4_, 0.005 g Na_2_S·9H_2_O, 1 g urea in 100 mL distilled H_2_O, pH 6.3–6.4), in between cycles, for 60 min, also under agitation (170 rpm), at room temperature. The specimens were then rinsed with distilled water for 5 s, before undergoing another erosive cycle. At the end of each day, the specimens were stored in artificial saliva, until the next cycle. The solutions were renewed after each cycle of the experiment and a new batch was prepared daily, with the pH and stability of the solutions being monitored daily.

### 2.3. Experimental Design

The forty-four artificially eroded teeth were randomly assigned to two different groups, depending on the adhesive system that was used: Clearfil™ SE Protect (Kuraray Noritake, Tokyo, Japan) as an experimental group (*n* = 22) and Clearfil™ SE Bond 2 (Kuraray Noritake, Tokyo, Japan) (*n* = 22) as the control group, both applied as a self-etch, following manufacturer’s recommendations (Table 2).

The two groups were then divided into four sub-groups, according to the type of test that was held, as well as its timing:

- Immediate (24 h) microtensile bond strength (*n* = 8);
- Immediate (24 h) hardness and elastic modulus (*n* = 3);
- Long-term (after 3 months of aging) microtensile bond strength (*n* = 8);
- Long-term (after 3 months of aging) hardness and elastic modulus (*n* = 3).

This division ultimately led to the following groups: CFSE_24–Clearfil SE Bond (24 h); CFSE_3M–Clearfil SE Bond (3 months); CFP_24–Clearfil Protect (24 h); and CFP_3M–Clearfil Protect (3 months).

The specimens tested after three months in storage were kept in artificial saliva, renewed weekly. The current guidelines of orientation from the Academy of Dental Materials (ADM) were followed to perform sample size calculation [28]. For microtensile bond strength testing, at least five samples are recommended and ideally eight samples [28]. In this study, for each adhesive system tested, sixteen samples were tested, eight immediately after restoration (24 h) and eight after water-storage (3 months). For hybrid layer hardness testing, for experimental reasons as it was an exploration analysis, only three samples were selected per experimental group.

### 2.4. Restorative Procedures

After demineralization and further allocation of specimens, the smear layer was simulated (600-grit SiC paper, Buehler Ltd., Lake Bluff, IL, USA), under running water for 60 s, using a polisher machine (LabolPol-4, Struers A/S, Ballerup, Denmark) (Armstrong et al., 2017). According to their experimental group, the teeth were treated as described in Table 2. The samples were immediately stored in artificial saliva and tested either after 24 h or 3 months.

**Figure 1:**
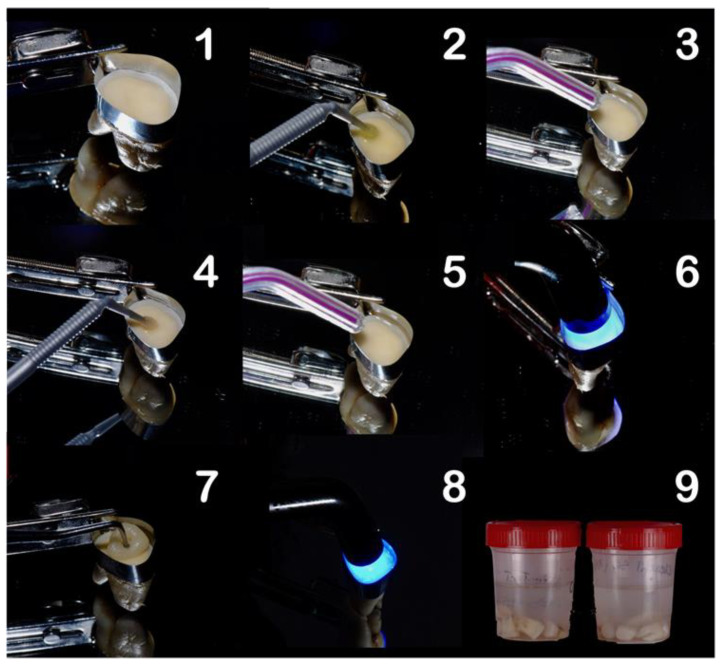
Restorative procedure of the experimental groups, where: (1) application of matrix band, (2) application of the primer, (3) drying step, (4) application of the bond (adhesive), (5) air thinning, (6) light-curing, (7) composite build-up, (8) light-curing in 2 mm increments, and (9) storage of the specimens.

Composite build-ups using Ceram.xSpectra™ ST HV (Dentsply DeTrey GmbH, Konstanz, Germany) were then constructed incrementally (three increments of 2 mm each), to cover all exposed dentinal surface and individually light-cured for 20 s each, at minimal tip distance (~0) using an LED light-curing unit (DB686, Froshan COXO Medical Instruments, Fuashon, Guangdong, China), at a mean irradiance at light exit of 700 mW/cm^2^, with a wavelength range of 420–480 nm. The light-curing unit was measured with an analog radiometer (Optilux radiometer SDS, Kerr, Orange, USA), after every three consecutive uses. Each sample was left with a 6 mm resin composite build-up, confirmed with a periodontal probe. The full restorative procedure is shown in Figure 1.

### 2.5. Microtensile Bond Strength (μTBS)

The specimens used to test microtensile bond strength (μTBS) were, after storage for 24 h or 3 months, sectioned longitudinally in the mesio-distal (X) and buccal-lingual (Y) directions, across the bonded interface, using a water-cooled diamond saw (Accutom-50, Struers A/S, Ballerup, Denmark). This process resulted in rectangular composite-dentin bonded sticks—excluding the composite-enamel bonded sticks—with a cross-sectional area of 1.0 mm^2^. The dimensions of each stick were measured using a digital caliper (Storm digital caliper, CDC/N 0 150 mm, Pontoglio, Italy) and recorded to calculate the bonded area.

These composite-dentin bonded sticks were afterwards attached to a Geraldeli type jig, using cyanoacrylate adhesive (Zapit, Dental Ventures of America, Corona, CA, USA) and tested under tensile stress in a universal testing machine (Shimadzu, Autograph AG-IS, Tokyo, Japan) until failure. The load cell used was 0.5 kN, with a crosshead speed of 0.5 mm/min. The μTBS (MPa) was calculated dividing the load at failure by the cross-sectional bonding area (mm^2^). To evaluate the failure modes, the fractures surfaces of the composite-dentin bonded sticks were evaluated using a stereo zoom microscope (EMZ-8TR, MeijiTechno Co., Ltd., Saitama, Japan) at 20× magnification. The failures were classified either as cohesive (failures that occurred exclusively within dentin—CD—or the composite build-up—CC), adhesive (failures that occurred exclusively at the composite/dentin interface–A) or mixed (failures that simultaneously occurred at composite/dentin interface and within dentin/composite–M). For statistical purposes, and following the ADM guidance, pre-testing failures (PTF) were included in the calculated means as 0 MPa.

### 2.6. Nanoindentation

Samples considered for nanoindentation were either immediately (24 h), or after 3 months, fixed in epoxy resin (Epofix Kit, Struers APS, Pederstrupvej, Denmark). After fixation, each sample was sectioned transversally, using a water-cooled diamond saw (Accutom-50, Struers A/S, Ballerup, Denmark), originating three slices per tooth (2 mm thickness).

Each slice of the sectioned tooth was then cleaned, air-dried and polished under water irrigation. For finishing and polishing, diamond grit polishers and trimmers, (10,000 rpm) were initially used (Henry Schein, CAD/CAM lab finishing and polishing Kit HP 900–9510, Melville, NY, USA) with a high-speed handpiece. Then, a soft and ultra-soft diamond paste was used, with two different soft bristle brushes, mounted in a low-speed handpiece (Diashine extraoral finishing and polishing system, 3M™ ESPE™ Lava™ Ultimate, St. Paul, MN, USA). To guarantee that fine polishing was accomplished, each sample was analyzed and observed under an endodontic optical microscope (Carl Zeiss Meditec AG, Jena, Germany), at 16× magnification.

After finishing/polishing sequences, the resin-eroded dentin slice interfaces (*n* = 3 for each group) were taken to a computer-controlled nanoindenter (NanoTest, Micro Materials Limited, Wrexham, UK) equipped with a Berkovich triangular pyramidal diamond indenter. After hydration of each specimen, transferal to the indenter was carried out using a computer-controller X-Y table. Nanoindentation experiments were performed in load control mode through the composite-adhesive-dentin interface regions, by selecting an area with the help of an optical microscope. For each sample, at least two matrices were programmed and performed: 12 rows × 8 columns (total of 96 indentations) or 11 rows × 9 columns (total of 99 indentations). The distance between rows was 3 or 4 μm, while the distance between columns was 5 μm. The indentations started in dentin and moved upwards across the hybrid layer (HL), the adhesive layer (AL) and the composite, using a maximum load of 1 mN. Loading/unloading was carried out in 5 s, with 2 s at maximum load. Hardness (H) and reduced Young’s modulus (Er) were calculated using the Oliver and Pharr’s analysis method [29].

### 2.7. Statistical Analysis

The experimental unit in this study was the tooth. To perform inferential hypothesis testing, the IBM SPSS Statistics software version v. 27.0 for Mac (IBM Corporation, Armonk, USA) was used. A two-way analysis of variance (ANOVA), considering as fixed factors: (1) the adhesive system and (2) the time point, was computed, in order to compare the effect of different adhesive systems over time, on the microtensile bond strength. For the mechanical properties (hardness/elastic modulus), a non-parametric alternative Kruskal–Wallis H Test (KW) was used as assumptions were not fully met for parametric tests. Post-hoc tests included Dunn’s test for KW. Although all failure modes obtained in microtensile bond strength testing were registered, only adhesive, mixed and PTF were considered in the statistical analysis, given that cohesive failures do not reflect a trustworthy value for bond strength. For all tests, a level of significance of 5% was set, accepting the null hypothesis if *p* ≥ 0.05.

## 3. Results

### 3.1. Microtensile Bond Strength and Fractographic Analysis

**Figure 2:**
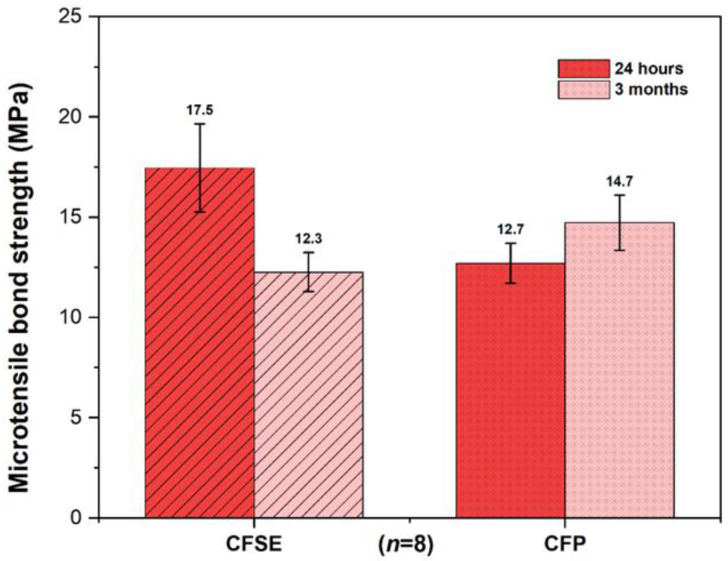
Bar chart comparing the experimental groups at different time points (immediate and after 3 months aging). No significant differences were found between the different experimental groups (ANOVA two-way, *p* = 0.087; *n* = 8). Error bars shown are standard error of the mean values (SE).

Results from the microtensile bond strength test are illustrated in Figure 2. No significant differences were found between the experimental groups at different time points, as evidenced by two-way ANOVA (*p* = 0.087), although an increasing trend was noted in the CFP_3M group. This contrasts with the decrease found in the CFSE_3M group. Additionally, a significant interaction was identified among the two factors analyzed (adhesive system and time point), (ANOVA, *p* = 0.025), shown in Table 3. This shows that the influence of time on the microtensile bond strength depends on whether CFSE or CFP is used. The observed effect size of the interaction is however weak (partial eta squared = 0.17).

Total counts, presented as percentages, of the different types of failures seen at the resin-dentin interface (adhesive, cohesive and mixed) are summarized in Table 4. The most predominant type of failure was the adhesive, in all groups, followed by mixed failures, in exception to CFSE_24 H group, which registered 27% cohesive failures in composite.

### 3.2. Hardness and Reduced Young’s Modulus

Hardness and reduced *Young’s* modulus values were assessed in three different layers: the hybrid layer, the adhesive layer and in dentin. These values are summarized in Table 5, according to the experimental group and time point. The distribution of H values was found to be significantly different in the hybrid layer (KW x^2^ (3): 11.1; *p* = 0.011), adhesive layer (KW x^2^ (3): 18.8; *p* < 0.001) and dentin (KW x^2^ (3): 19.9; *p* < 0.001). Regarding E, the distribution of the values was also significantly different in the hybrid layer (KW x^2^ (3): 8.5; *p* = 0.037), adhesive layer (KW x^2^ (3): 17.0; *p* = 0.001) and dentin (KW x^2^ (3): 15.7; *p* = 0.001).

#### Hardness and Reduced Young’s Modulus Maps

**Figure 3:**
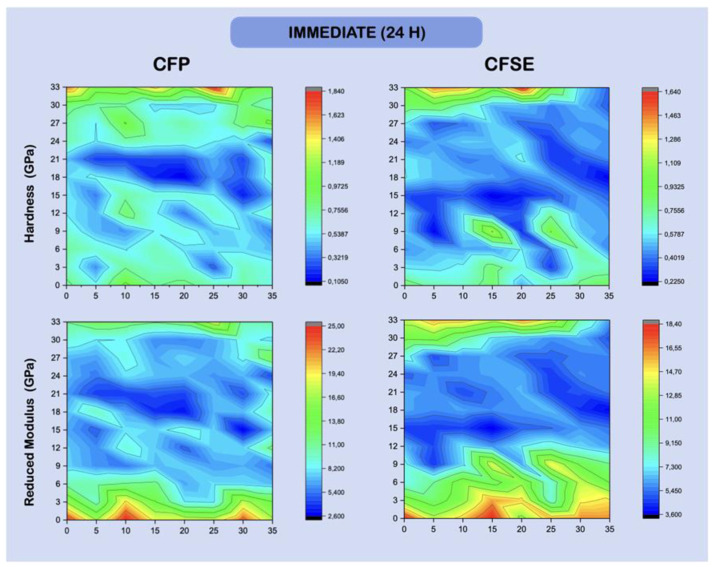
Hardness and reduced *Young’s* modulus (GPa) maps, showing the variation in intensity for each of the variables, across an area, mapping the resin-dentin interface after being restored and stored for 24 h in deionized water. Dentine is shown at the bottom, easily identifiable by the higher modulus values, while the hybrid layer is directly after (maps are representative of one random sample). Distance between points is shown in μm.

**Figure 4:**
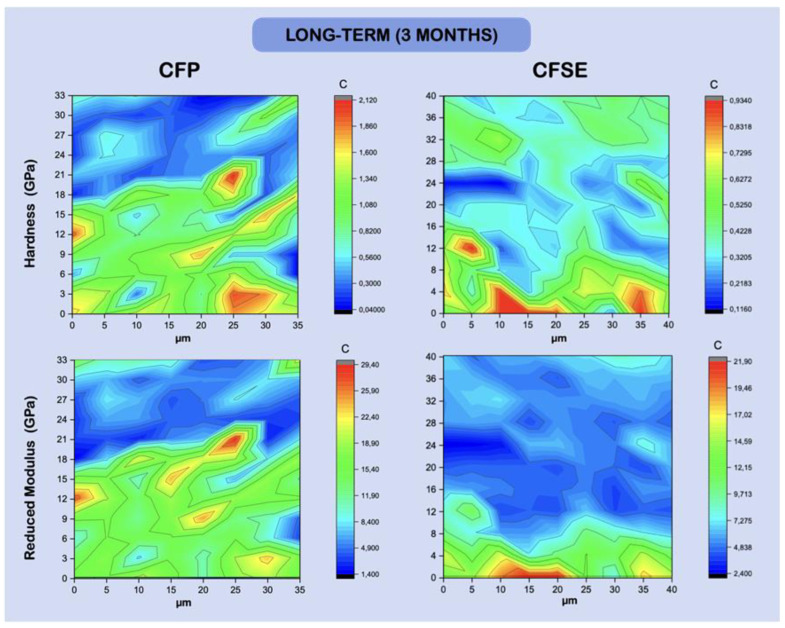
Hardness and reduced *Young’s* modulus (GPa) maps, showing the variation in intensity Figure 3. Random variation of hardness values can be seen in the CFSE group, while the reduced modulus shows distinguishable dentine features on the bottom layer (maps are representative of one random sample).

Maps illustrating the variation in hardness and reduced *Young’s* modulus across the resin-dentine interface, in a fixed matrix, are shown in Figure 3 and Figure 4. The HL and the AL show similar hardness and reduced modulus distribution, in the immediate and long-term results, and may not be distinguishable from each other, by examining the maps.

## 4. Discussion

The present laboratory study aimed to compare and evaluate the adhesive interface of a functional adhesive system when the substrate is eroded dentin. The results warrant acceptance of both null hypotheses, given that no statistical differences were observed between the groups and time points under research.

It is well known that adhesion to dentin remains the main challenge in adhesive dentistry [5,30]. In order to form a resin/collagen cohesive and crosslinked hybrid layer, the resin monomers must adequately infiltrate and co-polymerize within the exposed collagen mesh [7,8,31]. Not only does this required infiltration pose a challenge in an organic tissue like dentin [14,32,33], recent literature seems to agree that the quality of the adhesive bond becomes even more threatened in erosion [2,3,4,13,34,35]. The search for materials able to improve the longevity of contemporary restorations, specifically functional materials with novel molecules is still ongoing in present investigations [36,37].

Authors such as Siqueira et al. (2018) or Warreth et al. (2020) describe morphological and histological alterations on eroded dentin, capable of compromising the establishment of a cohesive hybrid layer [9,38]. In fact, Tay and Pashley (2004) and Ururahy et al. (2017) emphasize the presence of enlarged dentinal tubules, invaded by thick and porous peri-tubular dentin, which can lead to tubule occlusion and an overlying hypermineralized layer [10,39]. Considering these alterations, authors describe eroded dentin as a substrate with a negative impact on monomer infiltration [13,40], confirmed in laboratory studies where sound dentin demonstrated significantly higher bond strength results when compared to eroded dentin [16,34,40,41]. Regarding long-term bond strength results on eroded dentin, Flury et al. (2017) highlights that the hybrid layer is subject to accelerated degradation when compared to sound dentin. This was also proven by Siqueira et al., (2018) on a laboratory study, demonstrating that after a 2-year water-storage eroded specimens presented higher bond strength results if they were pre-treated with a cross-linking agent [9,41]. As speculated by Zimmerli et al. (2012) and Costa et al. (2019), these results were a consequence of the accelerated enzymatic and hydrolytic degradation of unpolymerized monomers and residual collagen fibers, thus contributing to a poor and less successful hybrid layer [40,42].

CFSE was chosen in this study as the control material as it currently represents the gold-standard adhesive when bonding to dentin, as proven in randomized clinical trials (2- and 8-year follow ups, respectively), and acknowledged in recent meta-analytical data [5,43,44]. This adhesive, introduced by Kuraray, benefits from a functional and original monomer–10-MDP–showing better bond strength results and longevity than any other formulation studied after the patent had expired [5,45]. By establishing a strong, cohesive and less soluble bond to calcium present and released from hydroxyapatite, after partial demineralization, this adhesive leads to the possibility of creating a nanolayering phenomenon, as described by Yoshihara et al., (2011) and Tian et al., (2016) [17,46]. Given these characteristics, CFSE is found to be responsible for high bond strength results, and a stable interface, both short- and long-term in dentin [5,47,48]. CFP, a more recent formulation, benefits from a similar composition as SE Bond. Additionally, another monomer was introduced to its composition–12-Methacryloyloxydodecyl pyridinium bromide (12-MDPB), a monomer containing a quaternary ammonium compound (QAC) chemical group, with a methacrylate group on the opposite end [49,50]. Given their respective composition, Clearfil SE Bond 2 is thus the ideal control when testing Clearfil SE Protect. 12-MDPB is described as a powerful antibacterial agent, able to co-polymerize within the organic matrix to other monomers [24,51,52,53], and provide an inhibitory action against oral Streptococci, without compromising the quality of the adhesive interface [15,23,50,54]. This antibacterial action has proven to be a promising strategy regarding treatment longevity, as proposed by Hashimoto et al. (2018) [55]. Moreover, Clearfil SE Protect benefits from long-term fluoride release from the functionalized reactive fluoride particles that it comprises [49]. Viana et al. (2020) suggest that fluoride release in the adhesive context can lead to fluorapatite precipitation [56]—a less soluble mineral—and thus leave dentin protected from further demineralization, as also described by Ayres et al. (2015) [57]. Furthermore, after new exposition to acidic challenges while in the presence of reactive fluoride particles, an acid-base resistant layer is formed (ABRZ), able to protect the underlying dentin, as was previously described in past research [21,22,58,59]. Tsuchiya et al. (2004) were able to microscopically analyze this layer, ultimately describing a resistant layer, which was located superiorly to the hybrid layer [60]. Given that this layer is material-dependent, as introduced by Shinohara et al. (2006), it was speculated that the functional adhesive CFP may gather the characteristics needed to protect the eroded substrate, promoting its mineralization and stability over time [61].

Microtensile bond strength and nanomechanical properties were tested both in short (24 h) and long-term (3 months). The calculation of nanohardness and Young’s elastic modulus provided clinically relevant information regarding the mechanical properties of the adhesive layers formed, highlighting clinically important phenomenon such as their potential resistance to wear and elastic deformation [62,63].

After immediate tensile and hardness testing (24 h), the samples were conserved in artificial saliva, simulating the same oral conditions in vivo and thus, the remineralization process, similarly to other studies conducted in the past [57].

Regarding microtensile bond strength tests on eroded dentin, results from the CFSE_24 were in agreement with current evidence. In fact, authors such as Deari et al. (2017) [64] or Ramos et al. (2013), [34] found similar mean results. Moreover, these results were significantly lower in contrast with the ones found in literature regarding sound dentin, which is also coherent, as shown by Francisconi-dos-Rios et al. (2015) or Deari et al. (2017) [64,65]. When compared to the results of CFP_24 (Figure 2 and Table 3), the mean values were not statistically different. Indeed, the differences between the two adhesives do not justify an immediate difference in bond strength results, given that their composition is almost identical. Furthermore, these values converge with past studies, in which other authors describe statistically similar immediate bond strength results in sound dentin, using the same two adhesives [61]. Given that CFP reached the same bond strength results when compared to the gold standard, we can theorize that the two different components present in the CFP does not seem to affect the immediate bond strength in eroded dentin. Thereby, CFP may present itself as a trustworthy option, as also confirmed by studies which evaluated this adhesive [55,58,61].

Bond strength results after aging also did not show significant differences. A decreasing trend with lower results after aging, when an adhesive system is tested after long-term storage in dentin, is not only expectable but certain, due to enzymatic and hydrolytic degradation [42,66,67]. This was seen with the CFSE_3M group. However, with CFP_3M, even though it was not statistically significant, a rise in bond strength was seen compared to the immediate result. These results echo the ones found in a study which also assessed the durability of CFP and found it to be stable over time [68]. Apparently, this group was less vulnerable to a hybrid layer breakdown, which is pointed out in the literature [15,23,50,54]. The accepted theory for this trend seems to be linked to 12-MDPB’s QAC group, which may be able to inhibit matrix metalloproteinases (MMPs) and thus, protect the hybrid layer, as previously investigated by Almahdy et al. (2012), Tezvergil-Mutluay et al. (2015), and Liu et al. (2011) [23,32,69]. It is hypothesized that MDPB can bind itself electrostatically to the negative charge of MMP active centers, thereby blocking their enzymatic action [70]. Activated by acidic environment, these endopeptidases are exposed during demineralization, becoming capable of hydrolyzing components of the extracellular organic matrix, leading to a breakdown of the hybrid layer, with loss of retention and the creation of gaps and water-rich zones [10,67]. This phenomenon seems to be especially promoted when the substrate is eroded [71]. Thus, a pre-treatment intervention aimed towards protecting the hybrid layer from MMPs would be useful [72]. Although in the last few decades the focus regarding MMP inhibition was concentrated in the use of chlorhexidine, namely 2%, [6,14,73,74], studies have lately been testing the incorporation of QACs in adhesive system formulations, alike in CFP [15,23].

Another factor that may have had an influence in the tendency for higher bond strength results in CFP_3M is the incorporation of reactive fluoride particles. In fact, authors like François (2020) have proven that these particles are capable of improving and enhancing the mechanical properties of the adhesive [75]. Moreover, the antibacterial and remineralization action that they may have, could be particularly useful when the substrate is eroded [76]. If successful, they could be reinforcing the underlying dentin, with resistant crystals and fluorapatite deposition along the adhesive interface, protecting the hybrid layer from premature degradation [24,25,51,76]. Considering these results and what is described in the literature up to this date, the functional adhesive CFP may be a promising strategy for long-term stability of eroded dentin, although studies with longer follow ups and a clinical setting are required.

Regarding failure analysis in microtensile bond strength testing, in all groups, the majority of the failures were adhesive (Table 4), both after 24 h and 3 months. These results are in fact predictable in this typology of testing that focused on CFP, as previously reported [23,49].

Considering the hardness and *Young’s* modulus results (Table 5), it is relevant to acknowledge that the integrity of the hybrid layer and its longevity are intimately related to its mechanical properties [47]. In fact, the hybrid layer’s elasticity is a key property that allows the substrate to absorb and withstand the forces received by the adjacent layers, such as the composite, as referred to by Illie et al. (2017) [63], while the material’s hardness is responsible to prevent wear [63,77]. The hardness and elastic modulus of the adhesive and hybrid layer showed lower results when compared to dentin, as expected and also found in other short-term [76,77] and long-term studies [11]. The hardness values differed between time points, when the adhesive layer was considered, for both groups, which may be consistent with degradation of the resin phase over time, due to hydrolytic damage. The same trend was seen with the elastic modulus reducing in the adhesive layer. These results are in fact predictable, being that the adhesive only differ by two components.

It was also possible to observe that the hardness values in dentin increased in the CFP group, with three months of aging. This could potentially be related to the formation of a reinforced remineralized layer, linked to the sodium fluoride particles reacting at the interface, also considering samples were stored in artificial saliva. In further acidic challenges, research has shown that a thicker ABRZ layer forms when CFP is used, compared to other adhesive strategies [27]. This is concentration-dependent and influences the resulting nanomechanical properties. Regarding the modulus, differences were also seen in the hybrid layer of the CFP_3M group, with a slight increase, becoming stiffer with aging, which may also be suggestive of the remineralization phenomenon. As for the rest of the modulus values and differences between groups, these followed the same trend seen with the hardness, as expected in these assays.

Although it is not possible to prove the enhancement of nanomechanical properties after three months of aging and possible remineralization, it is relevant to stress the fact that the bonding and mechanical properties of CFP were not only stable, but were also comparable to the gold standard control, as also previously described by Siqueira et al. (2020) [11]. Therefore, it would be important to extend the aging time in future studies while also exploring vibrational spectroscopy methods such as Fourier transform infrared and Raman spectroscopy, to characterize and map the chemistry of the resulting interface. This could confirm different interfacial chemistries and remineralization over time. To quantify the enzymatic activity and confirm its reduction, zymographic assays may also be planned.

Even though both null hypotheses were accepted, the increasing trend observed in the group that tested CFP seems to support the speculation that this adhesive may be able to prevent accelerated enzymatic degradation, proven to be a particular challenge in eroded dentin [71]. May this tendency be confirmed in studies with longer periods of storage (>5 years), it would be possible to conclude that CFP is able to prevent collagenolytic activity and form an acid-base resistant zone, thus protecting the hybrid layer and its longevity. The similarities in most of the properties are clearly linked to the fact that both adhesives share the same bulk composition and belong to a self-etch strategy with an application mode that is identical. The small differences and trends can thus be attributed to the functional monomer and the reactive particles, as described above, although longer aging periods may be required to observe larger differences.

## 5. Conclusions

Overall, based on the laboratory study conducted, and regarding bond strength, no differences were observed between the two adhesives tested, or between the two different time points. However, with CFP, a positive trend in microtensile values after aging was registered, which may be attributed to bond degradation resistance. Due to this, CFP could thereby be considered as a trustworthy strategy to improve bond longevity, even when compared to the current gold standard self-etch adhesive CFSE. When nanomechanical properties were considered, changes in dentin, such as an increase in hardness after 3 months when CFP was used and a small rise in elastic modulus of the hybrid layer with this adhesive, could also indicate remineralization phenomena occurring at the adhesive interface. Despite these differences, both CFSE and CFP showed very similar bonding and nanomechanical properties which can be explained by their similar chemical composition. Further studies are needed to elucidate the specific role of 12-MDP and the reactive filler in promoting MMP inhibition in eroded substrates and their subsequent remineralization.

## Figures and Tables

**Table 1:** List of restorative materials and their chemical composition according to information derived from the manufacturers.

Material	Manufacturer	Composition	
Clearfil™ SE Bond 2	Kuraray Noritake; Tokyo, Japan Batch number: 00128 Expiracy date: 31–03–2024	Primer: 10-MDP, HEMA, hydrophilic aliphatic dimethacrylate, dl-camphorquinone Solvent: water	Bond: 10-MDP, Bis-GMA, HEMA, hydrophobic aliphatic dimethacrylate, dl-camphorquinone, iniciators, accelarators, silanated colloidal silica
Clearfil™ SE Protect	Kuraray Noritake; Tokyo, Japan Batch number: 000070 Expiracy date: 30–09–2022	Primer: 10-MDP, MDPB, HEMA, hydrophilic dimetachrylate Solvent: water	Bond: 10-MDP, Bis-GMA, HEMA, hydrophobic dimethacrylate, di-canphorquinone, N,N-diethanol-p-toluidine, silanated colloidal silica, surface treated sodium fluoride
Ceram.xSpectra™ ST HV	Dentsply DeTrey GmbH, Konstanz, Germany Shade: A3 Batch number: 20110007000	Bis-EMA, Bis-GMA, UDMA, TEGDMA, dimethacrylate resin Glass fillers (78–80 wt%; 60–62% volume): ytterbium trifluoride, propylidynetrimethyl trimethacrylate, 1,12-dodecandioldimethacrylate, 2,6-di-tert-butyl-p-cresol	

10-MDP: 10-methacryloyloxydecyl dihydrogen phosphate; Bis-GMA: Bisphenol-A glycidyl dimethacrylate; Bis-EMA: Bisphenol A glycidyl methacrylate ethoxylated; HEMA: 2-hydroxyethyl methacrylate; MDPB; TEGDMA: triethylene glycol dimethacrylate; UDMA: urethane dimethacrylate.

**Table 2:** Adhesive type and adhesive strategy, step-by-step, used during sample preparation.

Adhesive	Self-Etch Strategy
Clearfil™ SE Bond 2 Clearfil™ SE Protect	Apply the primer on enamel and dentin, gently, for 20 s Without rinsing, gently dry with mild air flow for 5 s Apply a layer of bond on enamel and dentin for 20 s Make a uniform bond film using a gentle air flow Light-cure 10 s Place resin composite and light-cure for 20 s

**Table 3:** Two-way ANOVA results, considering the factors: experimental group and time point for the microtensile bond strength dependent variable.

Source	Type III Sum of Squares	d*f*	Mean Square	F	*p*
Model	135.142	3	45.047	2.419	0.087
Adhesive system	20.161	1	20.161	350.783	0.307
Time point	10.580	1	10.580	0.568	0.457
Adhesive system × time point	104.401	1	104.401	5.606	0.025
Error	521.412	28	18.622		
Total	7188.800	32			

**Table 4:** Summary of the fractographic analysis showing the different types of failures seen in each experimental group (presented as %). PTF indicates pre-testing failure–beams which fractured before bond strength testing was carried out.

Failures (%)		Adhesive	Cohesive Composite	Cohesive Dentin	Mixed	PTF
Immediate	CFSE	38	27	1	24	10
	CFP	58	14	1	17	11
3 months	CFSE	47	18	1	20	14
	CFP	52	14	1	19	13

**Table 5:** Hardness (*H*) and reduced *Young’s* modulus (*Er*) means (in GPa), and standard deviations shown between parenthesis, according to the adhesive (CFSE, CFP) and time point (24 h and 3 months). Different capital letters identify statistically significant differences within the same column, while different small letters identify significant differences within the same row (Dunn–Bonferroni, *p* < 0.05; *n* = 3).

		Immediate (24 h)			Long Term (3 m)		
		HL	AL	Dentin	HL	AL	Dentin
*H* (GPa)	CFSE	0.43 (0.19) ^aA^	0.62 (0.20) ^aA^	0.79 (0.23) ^aA^	0.32 (0.10) ^aA^	0.37 (0.19) ^bA^	0.76 (0.32) ^aA^
	CFP	0.52 (0.18) ^aA^	0.64 (0.21) ^aA^	0.83 (0.25) ^aA^	0.46 (0.30) ^aA^	0.39 (0.18)^bA^	1.52 (0.61) ^bA^
*Er* (GPa)	CFSE	6.08 (2.30) ^aA^	8.30 (3.07) ^aA^	13.91 (3.38) ^aA^	4.88 (1.07) ^aA^	5.61 (1.80) ^bA^	15.63 (5.27) ^aA^
	CFP	6.21 (1.13) ^aA^	8.17 (1.84) ^aA^	19.80 (4.39) ^aB^	6.44 (1.24) ^bA^	5.9 (1.10) ^bA^	19.44 (5.79) ^aA^

## Data Availability

The data presented in this study are available on reasonable request from the corresponding author.
